# Supplementary material for: Lime Rate in Clayey Soils Influences Chemical Fertility and Sugarcane Yield
Source: Plants (Basel). 2022 Aug 13;11(16):2110. doi: 10.3390/plants11162110 (PMC9415554; doi:10.3390/plants11162110)
Supplement: Supplementary file 1 [file plants-11-02110-s001.zip › plants-1836186-supplementary.pdf]

Supplemental Material:
Lime rate influences the chemical fertility and sugarcane yield in clayey soils

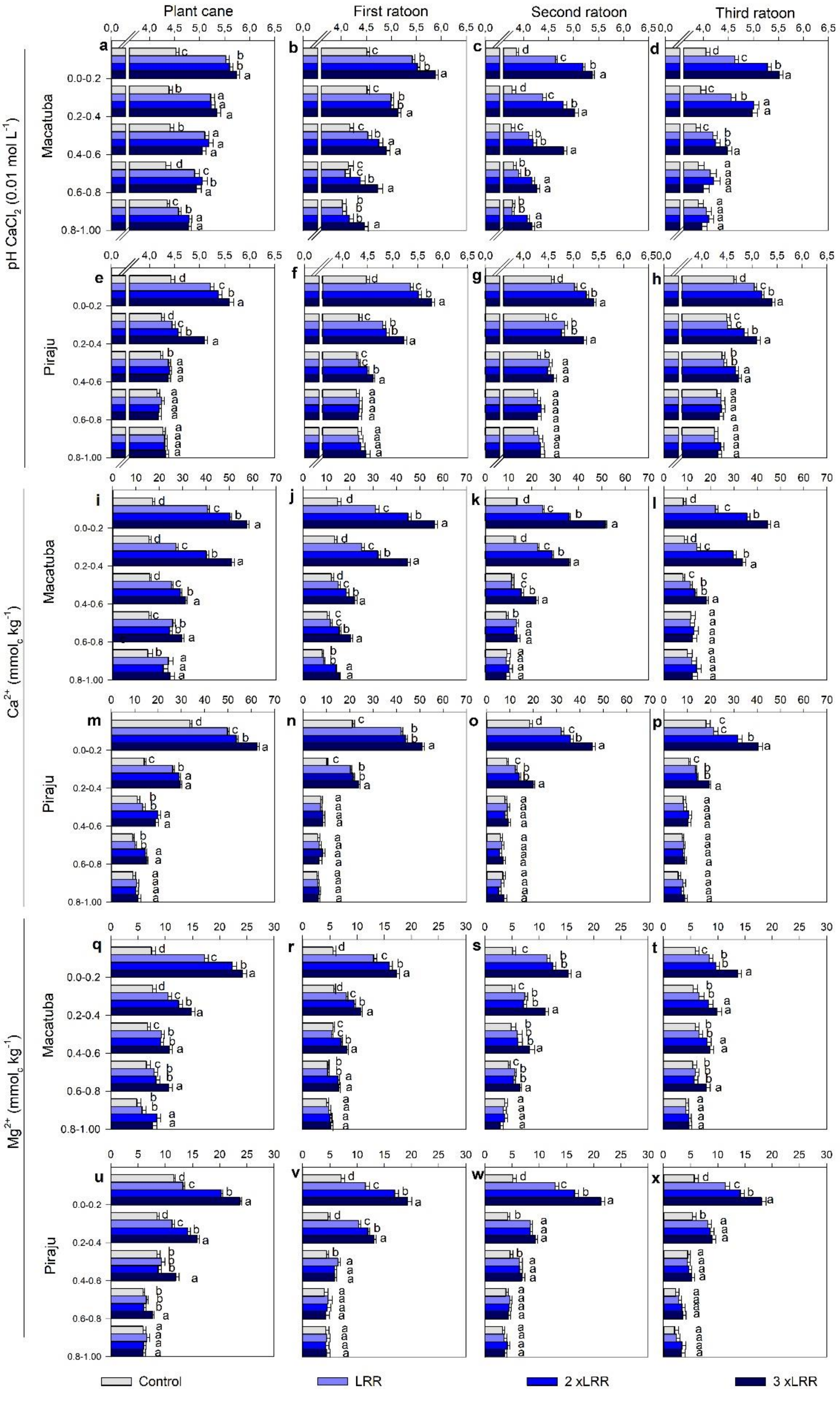

Figure S1. Soil pH$_{CaCl2}$ in Macatuba (a, b, c and d) and Piraju (e, f, g and h); soil concentration of Ca^{2+} in Macatuba (i, j, k, and l) and Piraju (m, n, o, and p) and Mg^{2+} in Macatuba (q, r, s, and t) and in Piraju (u, v, w, and x) across the soil profile under increasing lime rates on conventional soil tillage system (CT) for plant cane, first, second and third ratoon. Different lowercase letters indicate

significant differences between lime rates for the same depth and growing season (LSD, $p \leq 0.1$). Error bars express the standard error of the mean (n = 4).

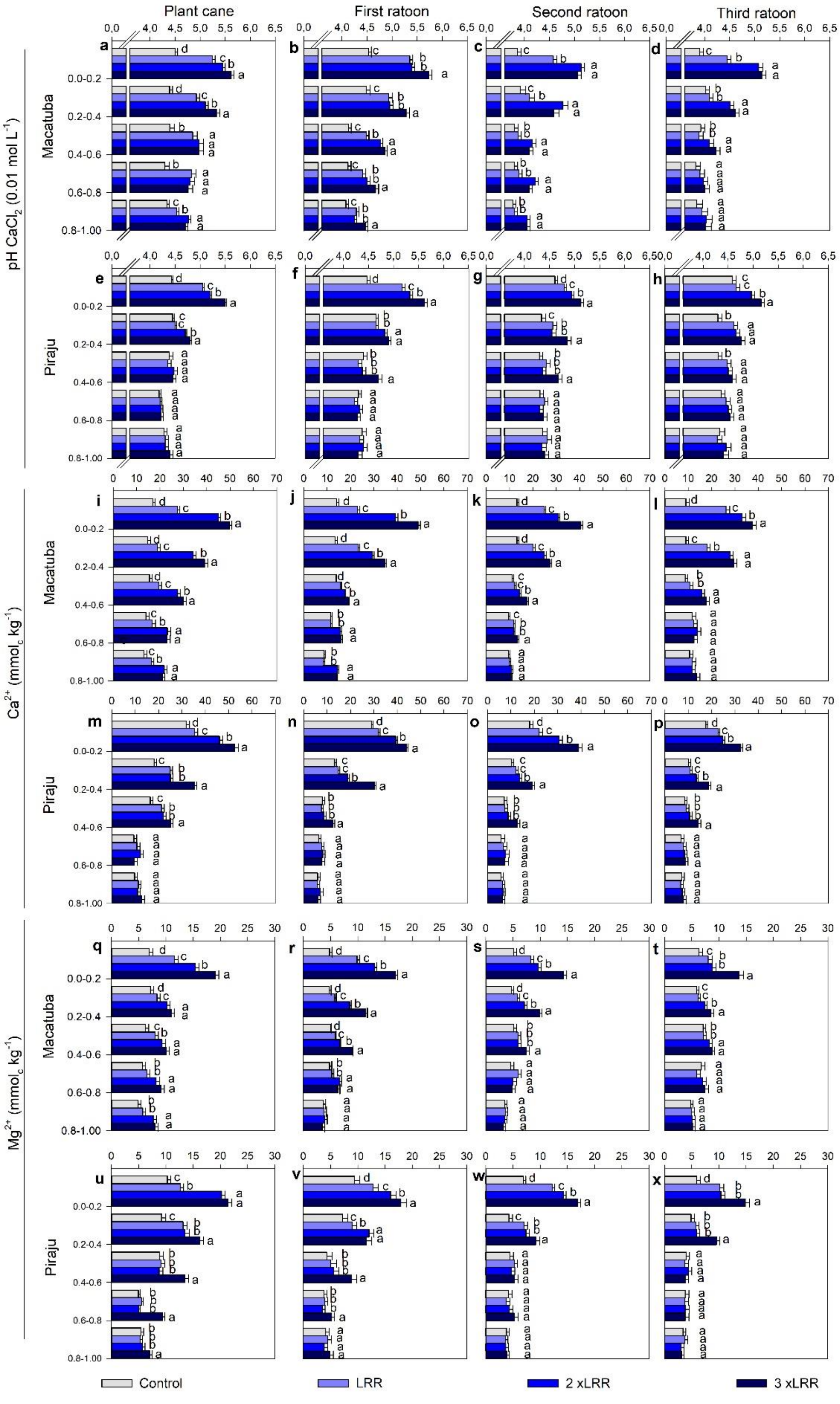

Figure S2. Soil pH_{CaCl2} in Macatuba (a, b, c and d) and Piraju (e, f, g and h); soil concentration of Ca^{2+} in Macatuba (i, j, k, and l) and Piraju (m, n, o, and p) and Mg^{2+} in Macatuba (q, r, s, and t) and in Piraju (u, v, w, and x) across the soil profile under increasing lime rates on deep-strip tillage system (DT) for plant cane, first, second and third ratoon. Different lowercase letters indicate significant differences between lime rates for the same depth and growing season (LSD, $p \leq 0.1$). Error bars express the standard error of the mean (n = 4).

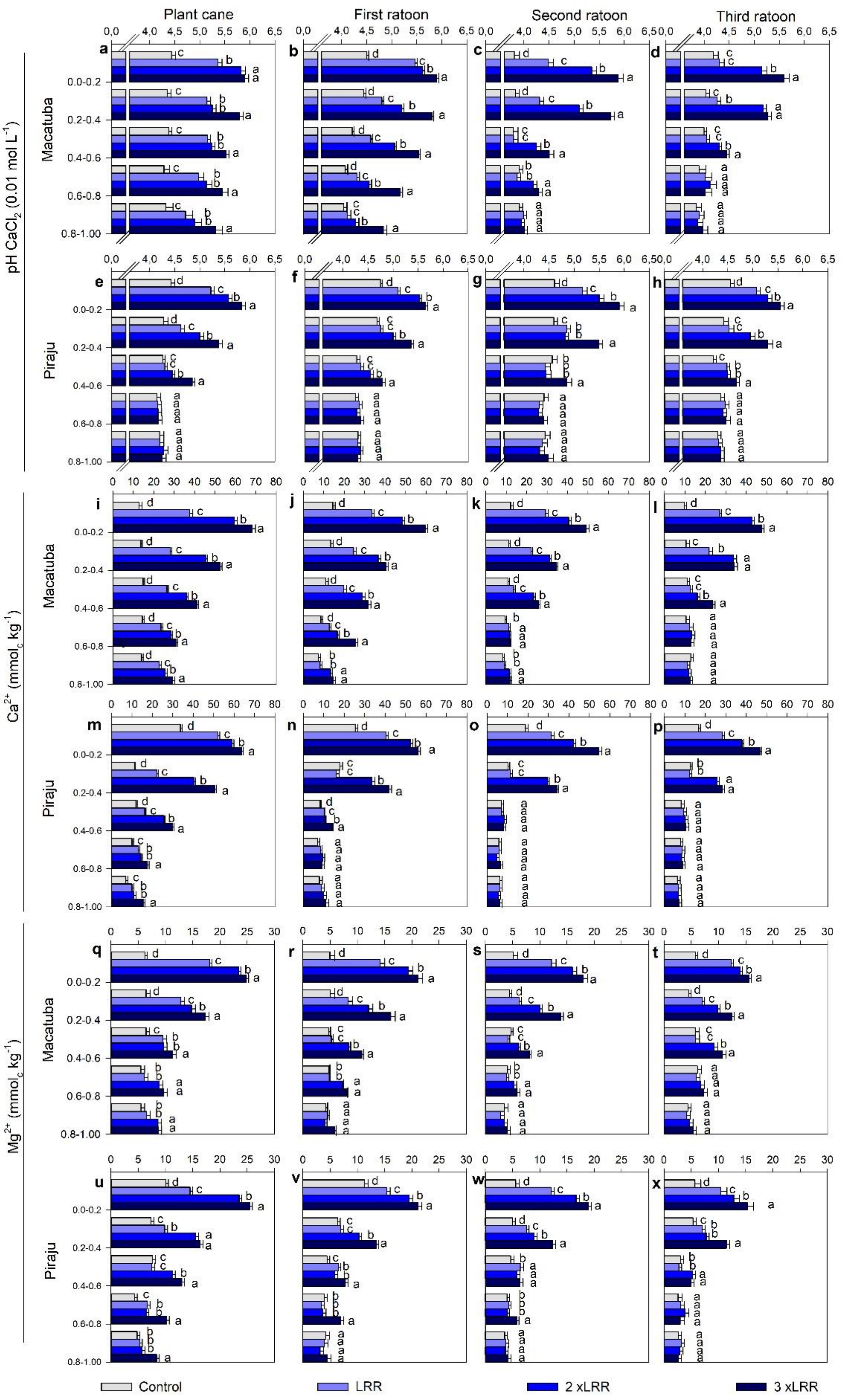

Figure S3. Soil pH$_{CaCl2}$ in Macatuba (a, b, c and d) and Piraju (e, f, g and h); soil concentration of Ca^{2+} in Macatuba (i, j, k, and l) and Piraju (m, n, o, and p) and Mg^{2+} in Macatuba (q, r, s, and t) and in Piraju (u, v, w, and x) across the soil profile under increasing lime rates on modified deep-strip tillage system (MDT) for plant cane, first, second and third ratoon. Different lowercase letters indicate significant differences between lime rates for the same depth and growing season (LSD, $p \leq 0.1$). Error bars express the standard error of the mean ($n = 4$).

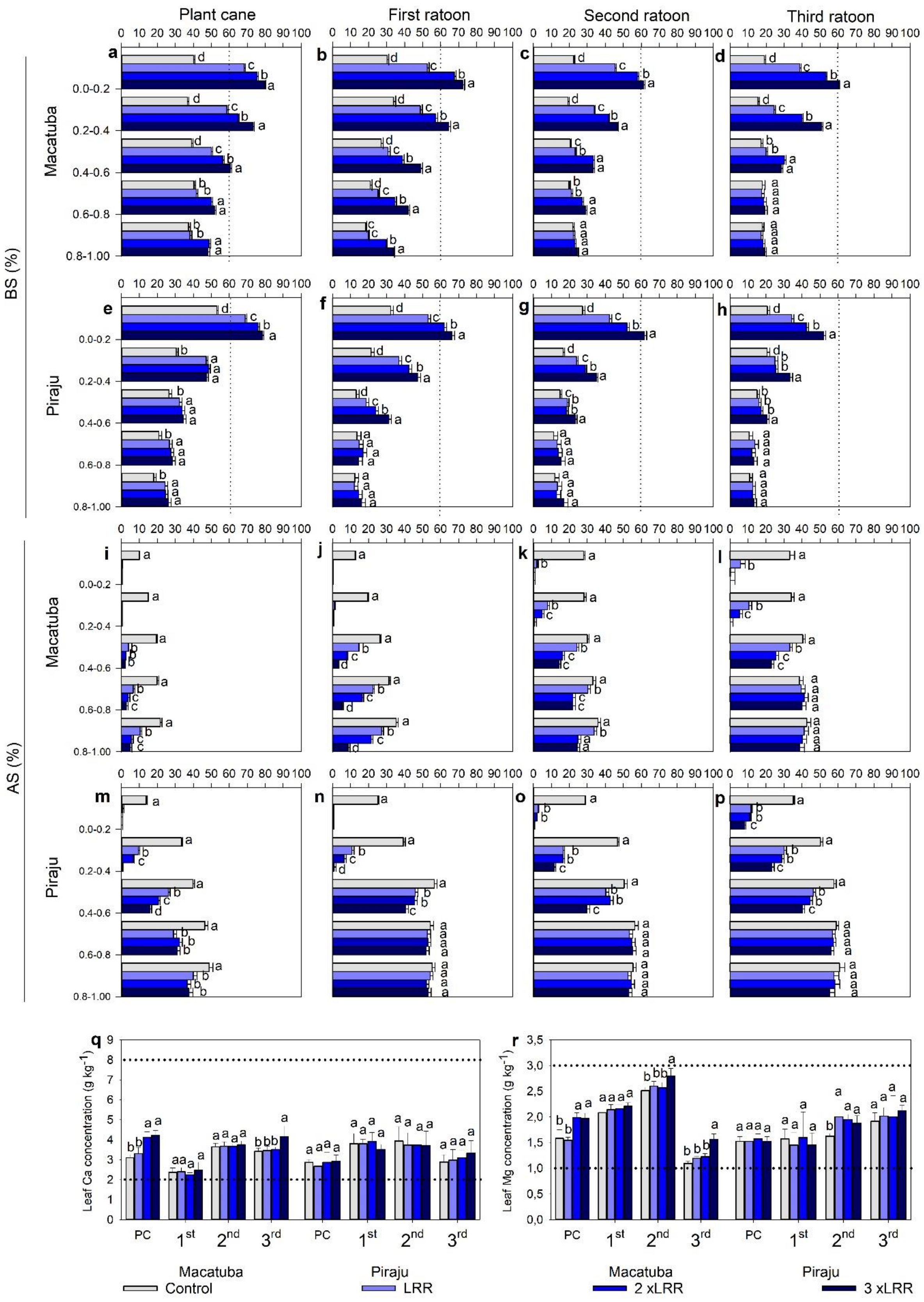

Figure S4. Base saturation in Macatuba (BS; a, b, c, and d) and Piraju (e, f, g, and h); Aluminum saturation in Macatuba (AS; i, j, k, and l) and Piraju (m, n, o, and p) across the soil profile under increasing lime rates and leaf concentration of calcium (Ca; q) and magnesium (Mg, r) in Macatuba and Piraju on conventional soil tillage system (CT) for plant cane, first, second and third ratoon. Different lowercase letters indicate significant differences between lime rates for the same depth and growing season (LSD, $p \leq 0.1$). Error bars express the standard error of the mean ($n = 4$).

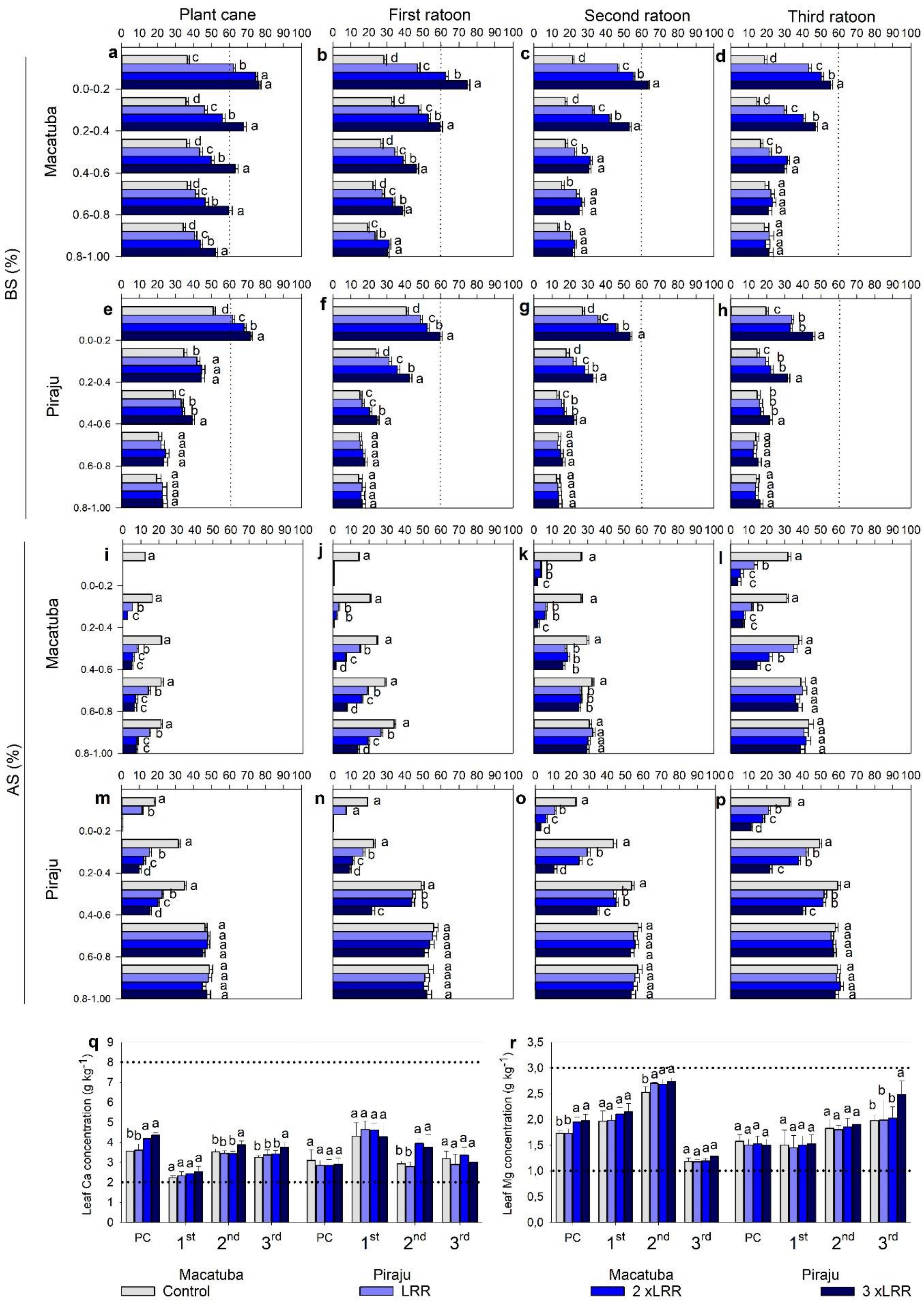

Figure S5. Base saturation in Macatuba (BS; a, b, c, and d) and Piraju (e, f, g, and h); Aluminum saturation in Macatuba (AS; i, j, k, and l) and Piraju (m, n, o, and p) across the soil profile under increasing lime rates and leaf concentration of calcium (Ca; q) and magnesium (Mg, r) in Macatuba and Piraju on deep-strip tillage system (DT) for plant cane, first, second and third ratoon. Different lowercase letters indicate significant differences between lime rates for the same depth and growing season (LSD, $p \leq 0.1$). Error bars express the standard error of the mean ($n = 4$).

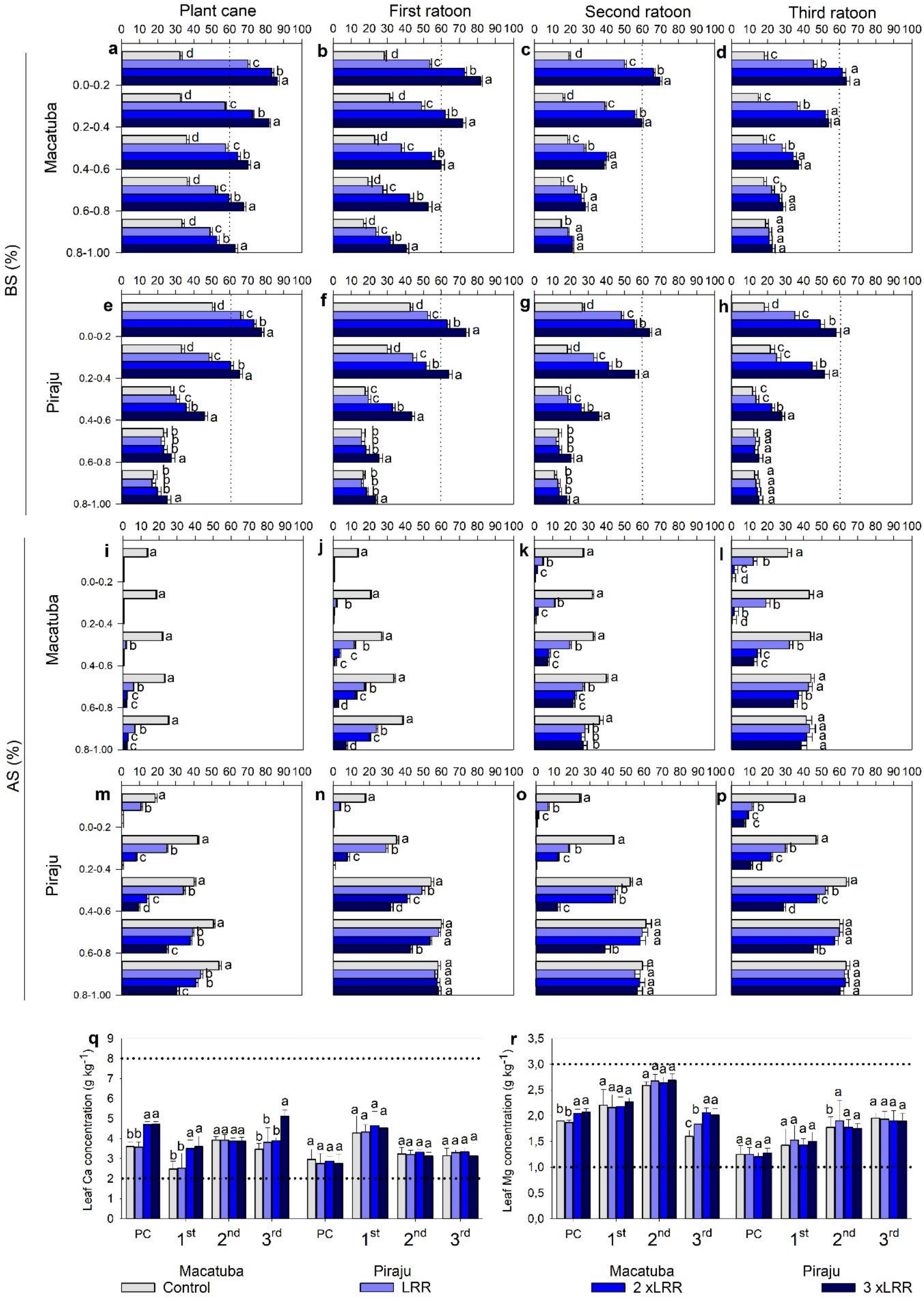

Figure S6. Base saturation in Macatuba (BS; a, b, c, and d) and Piraju (e, f, g, and h); Aluminum saturation in Macatuba (AS; i, j, k, and l) and Piraju (m, n, o, and p) across the soil profile under increasing lime rates and leaf concentration of calcium (Ca; q) and magnesium (Mg, r) in Macatuba and Piraju on modified deep-strip tillage system (MDT) for plant cane, first, second and third ratoon. Different lowercase letters indicate significant differences between lime rates for the same depth and growing season (LSD, $p \leq 0.1$). Error bars express the standard error of the mean ($n = 4$).

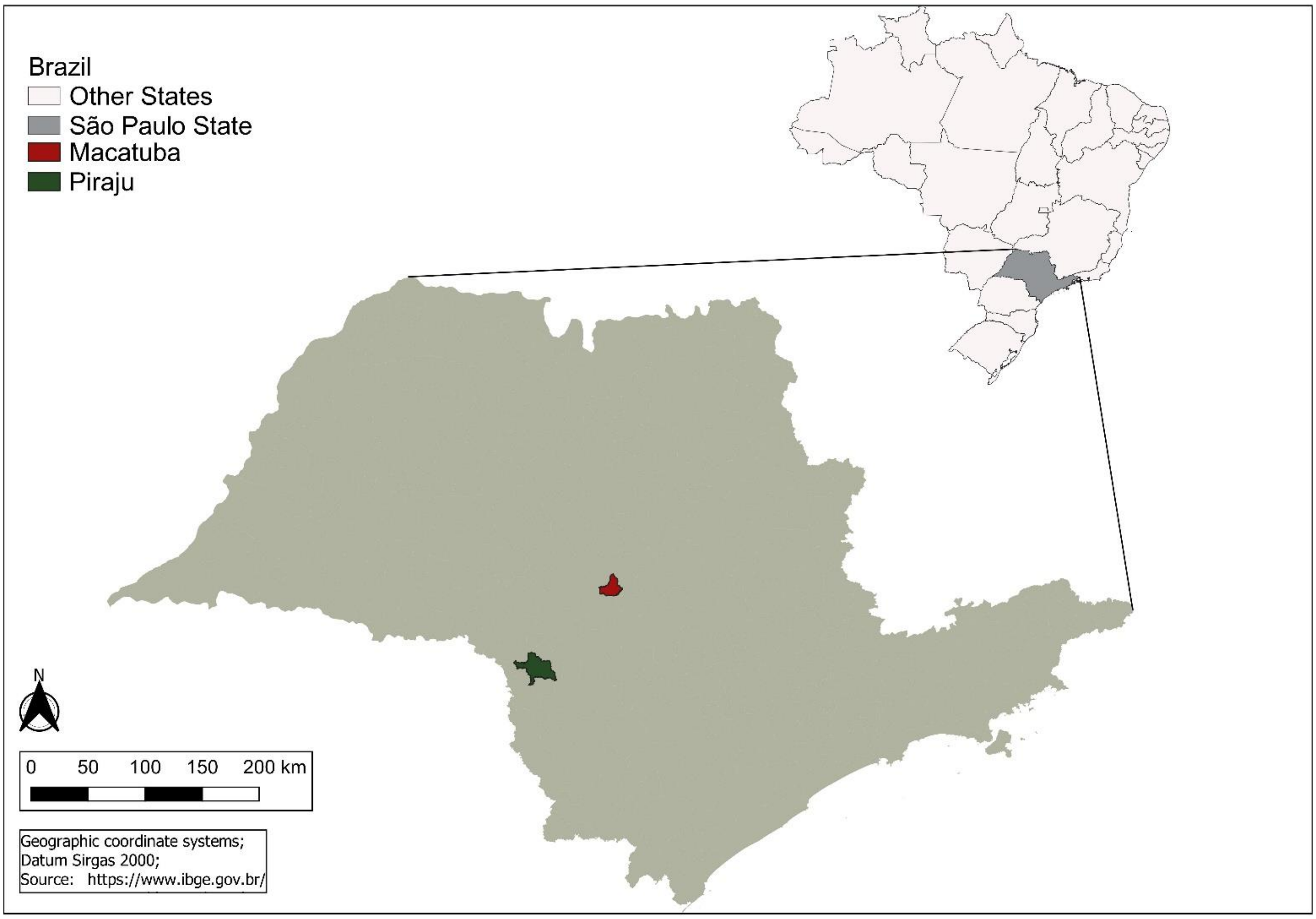

Figure S7. Geographic location of the experimental sites.

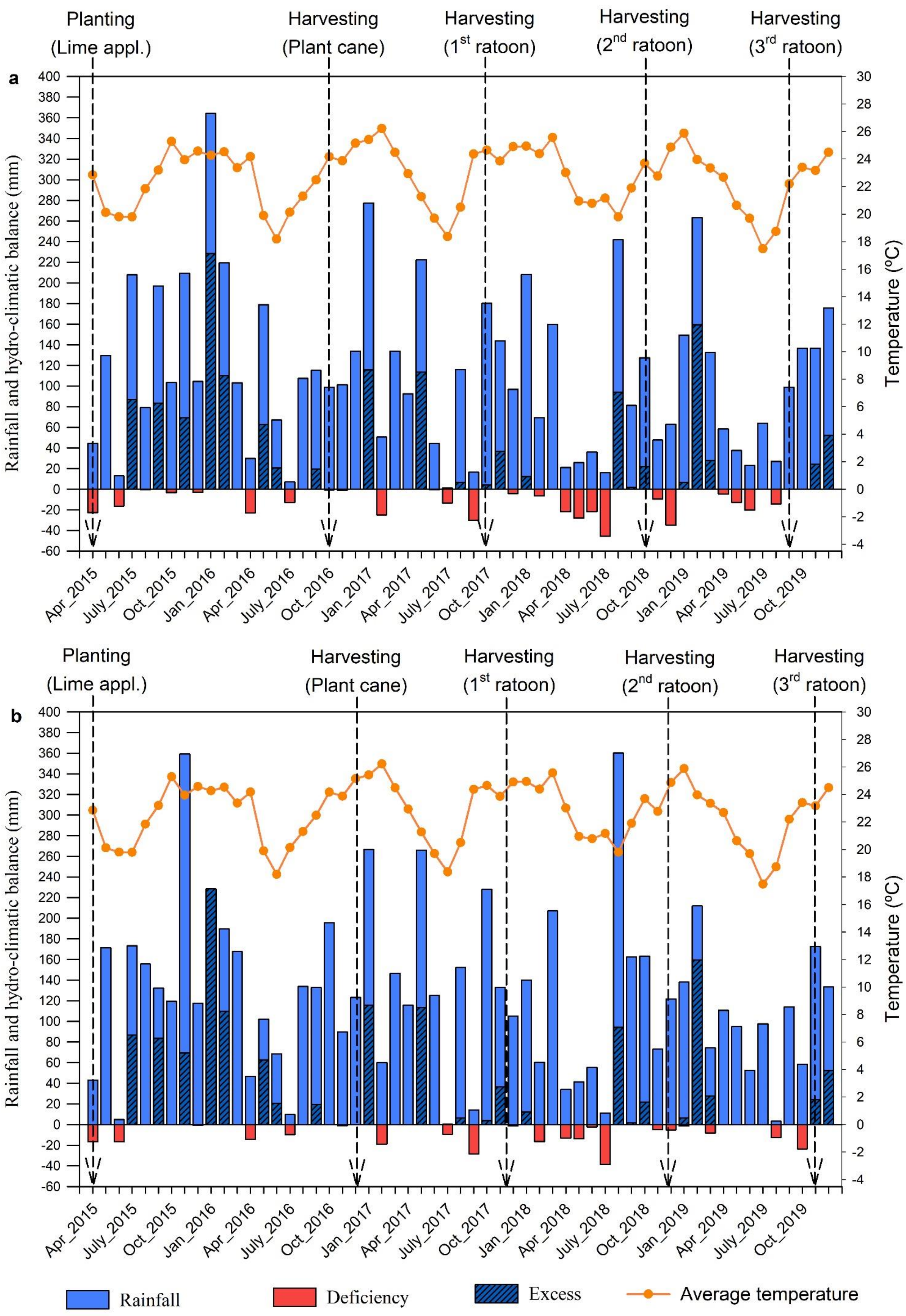

Figure S8. The average of monthly rainfall, temperature and hydro-climatic balance (mm) in the 2015/2019 growing season for (a) Macatuba and (b) Piraju.

Table S1. Average leaf concentration of each element in both tillage systems, growing seasons and locations

Elements	Average leaf concentration												
	CT[1]				DT[2]				MDT[3]				
	PC[4]	1st R[5]	2nd R[6]	3rd R[7]	PC	1st R	2nd R	3rd R	PC	1st R	2nd R	3rd R	RL[8]
	Macatuba												
N (g kg^{-1})	19.7	19.8	18.6	19.3	19.7	20.1	18.8	19.0	19.0	20.7	19.0	20.5	18-25
P (g kg^{-1})	1.8	1.9	2.0	1.8	1.7	1.8	2.0	1.9	1.8	1.9	2.0	1.9	1.5-3.0
K (g kg^{-1})	10.0	15.8	16.0	11.8	10.0	15.7	15.4	11.7	10.2	16.5	15.2	12.2	10-16
Ca (g kg^{-1})	3.7	2.4	3.7	3.6	3.9	2.4	3.6	3.4	4.1	3.0	3.9	4.1	2.0-8.0
Mg (g kg^{-1})	1.8	2.1	2.6	1.3	1.8	2.0	2.7	1.2	2.0	2.2	2.6	1.9	1.0-3.0
S (g kg^{-1})	1.6	1.8	1.8	1.7	1.6	1.7	1.7	1.8	1.6	1.7	1.7	1.8	1.5-3.0
Fe (mg kg^{-1})	86.3	85.2	103.6	104.7	81.9	90.5	115.5	114.2	83.5	85.1	106.1	114.8	40-250
Cu (mg kg^{-1})	6.7	9.1	6.7	12.7	5.8	9.0	6.7	12.3	6.1	9.6	6.8	12.5	6.0-15
Zn (mg kg^{-1})	19.4	20.4	12.4	29.1	18.0	20.8	16.1	29.8	18.5	21.4	16.1	33.8	10-50
Mn (mg kg^{-1})	89.0	84.3	51.1	70.3	91.3	78.5	52.4	78.2	100.2	84.2	50.4	72.7	25-250
B (mg kg^{-1})	11.0	13.2	13.3	12.8	11.1	13.3	13.9	13.0	11.5	12.9	13.6	13.2	10-30
	Piraju												
N (g kg^{-1})	19.3	20.3	18.7	20.6	19.4	20.2	19.2	18.8	19.5	20.6	19.1	18.7	18-25
P (g kg^{-1})	1.8	2.1	2.2	2.0	1.8	2.1	2.0	1.8	1.9	2.1	2.2	2.4	1.5-3.0
K (g kg^{-1})	9.9	11.9	9.8	10.8	9.8	11.2	9.7	10.6	9.7	11.7	10.5	11.4	10-16
Ca (g kg^{-1})	2.5	3.3	3.3	2.7	2.6	3.8	3.0	2.8	2.5	3.9	3.0	3.1	2.0-8.0
Mg (g kg^{-1})	1.5	1.5	1.9	2.1	1.5	1.5	1.8	2.1	1.3	1.5	1.7	1.9	1.0-3.0
S (g kg^{-1})	1.2	1.0	1.1	0.9	1.1	1.0	1.0	0.9	1.1	1.0	1.3	1.0	1.5-3.0
Fe (mg kg^{-1})	46.6	63.8	103.9	83.9	50.2	58.7	97.0	94.2	50.5	63.2	101.9	96.7	40-250
Cu (mg kg^{-1})	5.9	8.7	7.7	8.0	5.5	8.6	6.8	7.9	5.2	9.9	8.6	8.1	6.0-15
Zn (mg kg^{-1})	20.9	24.5	16.8	21.8	20.9	22.8	16.2	21.0	21.1	22.1	17.3	20.9	10-50
Mn (mg kg^{-1})	34.5	37.1	39.0	41.0	34.3	33.3	30.6	41.1	36.3	35.0	34.5	41.8	25-250
B (mg kg^{-1})	15.1	20.6	14.3	18.1	16.6	19.8	12.8	18.3	16.0	22.1	21.0	17.8	10-30

[1] Conventional soil tillage system; [2] Deep strip-tillage system; [3] Modified deep strip-tillage system; [4]Plant cane; [5]First ratoon; [6]Second ratoon; [7]Third ratoon; [8]Reference values of the concentration of each element.

Table S2. Principal component analysis for soil fertility parameters and the concentration of calcium (Ca^{2+}) and magnesium (Mg^{2+}) for conventional soil tillage system (CT)

		Eigenvalue	Total Variance %	Cumulative eigenvalues	Cumulative %
Macatuba	1	1.98	99.08	1.98	99.08
	2	0.02	0.92	2.00	100.00
Piraju	1	1.97	98.53	1.97	98.53
	2	0.03	1.47	2.00	100.00
		Eigenvectors		Correlations	
Active[2]		Factor1	Factor 2	Factor 1	Factor 2
Macatuba	St	0.707	0.707	0.995	0.096
	Sg	0.707	-0.707	0.995	-0.096
Piraju	St	0.707	0.707	0.993	0.121
	Sg	0.707	-0.707	0.993	-0.121
		Correlations between soil properties[1]			
	Macatuba		Piraju		
Supplementary[3]	Factor 1	Factor 2	Factor 1	Factor 2	
BS_1	0.90	-0.14	0.93	0.19	
BS_2	0.94	-0.12	0.91	0.19	
BS_3	0.88	0.30	0.76	0.52	
BS_4	0.78	0.44	0.72	0.60	
BS_5	0.78	0.45	0.73	0.52	
AS_1	-0.76	0.37	-0.88	0.17	
AS_2	-0.82	0.36	-0.93	0.18	
AS_3	-0.95	0.03	-0.76	-0.36	
AS_4	-0.86	-0.24	-0.82	-0.47	
AS_5	-0.92	-0.27	-0.85	-0.34	
Ca_1	0.82	-0.25	0.87	0.14	
Ca_2	0.82	-0.23	0.85	0.27	
Ca_3	0.88	0.21	0.44	0.58	
Ca_4	0.75	0.36	0.40	0.67	
Ca_5	0.65	0.39	0.30	0.76	
Mg_1	0.86	-0.05	0.79	-0.12	
Mg_2	0.83	0.04	0.87	0.12	
Mg_3	0.66	0.17	0.76	0.41	
Mg_4	0.66	0.23	0.62	0.51	
Mg_5	0.54	0.31	0.64	0.58	

[1]Correlations ≥|0.70| are significant (Manly. 1994). [2]Actives: Stalk yield (St); and Sugar yield (Sg). [3]Supplementary: Base and Aluminum saturation (BS and AS, %), and exchangeable calcium and magnesium concentration (Ca^{2+} and Mg^{2+}, $mmol_c$ kg^{-1}). The numbers 1, 2, 3, 4, and 5 represents the soil layers 0.0-0.2, 0.2-0.4, 0.4-0.6, 0.6-0.8, and 0.8-1.00 m, respectively.

Table S3. Principal component analysis for soil fertility parameters and the concentration of calcium (Ca^{2+}) and magnesium (Mg^{2+}) for deep-strip tillage system (DT)

		Eigenvalue	Total Variance	Cumulative eigenvalues	Cumulative
			%		%
Macatuba	1	1.97	98.59	1.97	98.59
	2	0.03	1.41	2.00	100.00
Piraju	1	1.97	98.29	1.97	98.29
	2	0.03	1.71	2.00	100.00
		Eigenvectors		Correlations	
Active[2]		Factor1	Factor 2	Factor 1	Factor 2
Macatuba	St	0.707	0.707	0.993	0.119
	Sg	0.707	-0.707	0.993	-0.119
Piraju	St	0.707	0.707	0.991	0.131
	Sg	0.707	-0.707	0.991	-0.131
		Correlations between soil properties[1]			
	Macatuba		Piraju		
Supplementary[3]	Factor 1	Factor 2	Factor 1	Factor 2	
BS_1	0.90	-0.02	0.93	0.24	
BS_2	0.95	-0.05	0.90	0.30	
BS_3	0.82	0.41	0.67	0.62	
BS_4	0.71	0.55	0.63	0.51	
BS_5	0.66	0.62	0.60	0.68	
AS_1	-0.90	0.19	-0.93	0.33	
AS_2	-0.88	0.24	-0.93	0.13	
AS_3	-0.93	-0.08	-0.77	-0.40	
AS_4	-0.81	-0.38	-0.70	-0.48	
AS_5	-0.80	-0.48	-0.62	-0.48	
Ca_1	0.83	-0.12	0.88	0.13	
Ca_2	0.84	-0.12	0.79	0.48	
Ca_3	0.81	0.42	0.48	0.72	
Ca_4	0.61	0.58	0.43	0.68	
Ca_5	0.49	0.63	0.35	0.77	
Mg_1	0.83	0.07	0.89	0.00	
Mg_2	0.79	0.14	0.86	0.29	
Mg_3	0.49	0.23	0.64	0.46	
Mg_4	0.33	0.37	0.53	0.38	
Mg_5	0.37	0.58	0.62	0.42	

[1]Correlations ≥|0.70| are significant (Manly. 1994). [2]Actives: Stalk yield (St); and Sugar yield (Sg). [3]Supplementary: Base and Aluminum saturation (BS and AS, %), exchangeable calcium and magnesium concentration (Ca^{2+} and Mg^{2+}, $mmol_c$ kg^{-1}). The numbers 1, 2, 3, 4, and 5 represents the soil layers 0.0-0.2, 0.2-0.4, 0.4-0.6, 0.6-0.8, and 0.8-1.00 m, respectively.

Table S4. Principal component analysis for soil fertility parameters and the concentration of calcium (Ca^{2+}) and magnesium (Mg^{2+}) for modified deep-strip tillage system (MDT)

		Eigenvalue	Total Variance %	Cumulative eigenvalues	Cumulative %
Macatuba	1	1.98	99.07	1.98	99.07
	2	0.02	0.93	2.00	100.00
Piraju	1	1.95	97.59	1.95	97.59
	2	0.05	2.41	2.00	100.00
		Eigenvectors		Correlations	
Active[2]		Factor1	Factor 2	Factor 1	Factor 2
Macatuba	St	0.707	0.707	0.995	0.096
	Sg	0.707	-0.707	0.995	-0.096
Piraju	St	0.707	0.707	0.988	0.155
	Sg	0.707	-0.707	0.988	-0.155
		Correlations between soil properties[1]			
	Macatuba		Piraju		
Supplementary[3]	Factor 1	Factor 2	Factor 1	Factor 2	
BS_1	0.93	0.11	0.88	0.11	
BS_2	0.80	-0.07	0.88	0.17	
BS_3	0.84	0.14	0.75	0.46	
BS_4	0.86	0.10	0.61	0.59	
BS_5	0.81	-0.01	0.52	0.69	
AS_1	-0.91	0.17	-0.96	0.01	
AS_2	-0.87	0.18	-0.97	-0.02	
AS_3	-0.85	-0.15	-0.92	-0.22	
AS_4	-0.84	-0.29	-0.73	-0.49	
AS_5	-0.86	-0.15	-0.61	-0.60	
Ca_1	0.94	0.12	0.78	0.06	
Ca_2	0.81	0.00	0.79	0.10	
Ca_3	0.66	0.44	0.76	0.32	
Ca_4	0.60	0.62	0.59	0.54	
Ca_5	0.64	0.22	0.35	0.71	
Mg_1	0.94	-0.03	0.81	0.13	
Mg_2	0.87	0.13	0.77	0.18	
Mg_3	0.79	0.21	0.55	0.22	
Mg_4	0.76	0.12	0.43	0.34	
Mg_5	0.65	0.25	0.34	0.53	

[1]Correlations ≥|0.70| are significant (Manly. 1994). [2]Actives: Stalk yield (St); and Sugar yield (Sg). [3]Supplementary: Base and Aluminum saturation (BS and AS, %), exchangeable calcium and magnesium concentration (Ca^{2+} and Mg^{2+}, $mmol_c\ kg^{-1}$). The numbers 1, 2, 3, 4, and 5 represents the soil layers 0.0-0.2, 0.2-0.4, 0.4-0.6, 0.6-0.8, and 0.8-1.00 m, respectively.

Table S5. Soil chemical attributes and texture characterization prior to the experiment

	Macatuba Depth (m)					Piraju Depth (m)				
Atributes	0.0-0.2	0.2-0.4	0.4-0.6	0.6-0.8	0.8-1.00	0.0-0.2	0.2-0.4	0.4-0.6	0.6-0.8	0.8-1.00
pH ($CaCl_2$)	4.4	4.0	4.0	3.9	4.0	5.0	4.5	4.2	4.2	4.2
OC (g kg^{-1})	23	15	12	10	10	19	16	11	9	9
P_{resin} (mg kg^{-1})	12	7	4	3	4	8	6	2	2	2
K ($mmol_c$ kg^{-1})	2.0	0.8	0.3	0.2	0.7	1.2	0.7	0.1	0.1	0.1
Ca ($mmol_c$ kg^{-1})	19	10	6	7	6	33	17	5	5	5
Mg ($mmol_c$ kg^{-1})	9	5	3	3	2	15	9	3	4	3
S (mg kg^{-1})	21	47	36	50	45	8	18	55	74	68
Fe (mg kg^{-1})	23	15	8	7	7	30	27	13	12	7
Cu (mg kg^{-1})	3	3.3	2.2	1.8	1.8	1.1	1.2	0.9	0.7	0.6
Mn (mg kg^{-1})	9.7	4.3	3	1.2	2	3.6	2.5	0.3	0.3	0.2
Zn (mg kg^{-1})	1.1	0.5	0.1	0.1	0.1	0.4	0.3	0.1	0.1	0.1
B (mg kg^{-1})	0.2	0.2	0.1	0.1	0.1	0.3	0.2	0.2	0.1	0.1
Al ($mmol_c$ kg^{-1})	5	7	8	7	8	0.7	6	10	11	10
H+Al ($mmol_c$ kg^{-1})	52	62	64	70	64	45	64	69	71	72
CEC ($mmol_c$ kg^{-1})*	82	78	73	80	73	94	91	76	80	78
Base saturation (%)**	37	20	13	13	12	52	28	11	11	9
Al saturation (%)***	14	30	46	40	48	2	27	62	62	64
Clay content (g kg^{-1})	358	449	486	469	442	673	735	734	757	746
Silt content (g kg^{-1})	154	140	116	124	146	129	93	99	85	92
Sand content (g kg^{-1})	488	410	398	407	412	198	173	167	158	163

*Calculated from the equation: CEC (mmo_c kg^{-1}) = Ca^{2+} + Mg^{2+} + K^{+} + (H+Al); ** Base saturation (%) = (Ca^{2+} + Mg^{2+} + K^{+}) / (CEC) x 100; ***Al saturation (%) = (Al / (Ca^{2+} + Mg^{2+} + K^{+} + Al^{3+})) x 100.
